# Identification of Nonlinear Soil Properties from Downhole Array Data Using a Bayesian Model Updating Approach

**DOI:** 10.3390/s22249848

**Published:** 2022-12-14

**Authors:** Farid Ghahari, Fariba Abazarsa, Hamed Ebrahimian, Wenyang Zhang, Pedro Arduino, Ertugrul Taciroglu

**Affiliations:** 1 Department of Civil & Environmental Engineering, University of California, Los Angeles, CA 90095, USA; 2 Department of Civil & Environmental Engineering, University of Nevada, Reno, NV 89557, USA; 3 Texas Advanced Computing Center, Austin, TX 78758, USA; 4 Department of Civil and Environmental Engineering, University of Washington, Seattle, WA 98195, USA

**Keywords:** nonlinear soil properties, geotechnical arrays, Bayesian estimation, earthquake data, inverse problem

## Abstract

An accurate seismic response simulation of civil structures requires accounting for the nonlinear soil response behavior. This, in turn, requires understanding the nonlinear material behavior of in situ soils under earthquake excitations. System identification methods applied to data recorded during earthquakes provide an opportunity to identify the nonlinear material properties of in situ soils. In this study, we use a Bayesian inference framework for nonlinear model updating to estimate the nonlinear soil properties from recorded downhole array data. For this purpose, a one-dimensional finite element model of the geotechnical site with nonlinear soil material constitutive model is updated to estimate the parameters of the soil model as well as the input excitations, including incident, bedrock, or within motions. The seismic inversion method is first verified by using several synthetic case studies. It is then validated by using measurements from a centrifuge test and with data recorded at the Lotung experimental site in Taiwan. The site inversion method is then applied to the Benicia–Martinez geotechnical array in California, using the seismic data recorded during the 2014 South Napa earthquake. The results show the promising application of the proposed seismic inversion approach using Bayesian model updating to identify the nonlinear material parameters of in situ soil by using recorded downhole array data.

## 1. Introduction

The near-surface soil layers are susceptible to nonlinear response behavior under moderate and strong earthquakes [1]. Although the thickness of nonlinear soil layers can be negligible compared with the path that seismic waves travel from the source to the site, they can have a significant contribution to the ground surface response [2]. Therefore, an accurate seismic response simulation of civil structures requires accounting for the nonlinear soil response behavior. This, in turn, requires understanding the nonlinear behavior of the in situ soil under real-life earthquake excitations.

Several analytical and numerical methods exist for site response analysis given the bedrock or outcrop motions (e.g., [3,4,5]). Nevertheless, their accuracy inherently depends on the properties of the soil layers, which are often obtained from lab testing disturbed samples. This is why the in situ soil behavior can be different from those observed in the lab. To resolve this problem, several field-testing methods exist to estimate in situ soil properties. Cross-hole tests (CHT) [6], seismic reflection [7], downhole tests (DHT), seismic refraction [8], suspension logging [9], and spectral analysis of surface waves (SASWs) [10] are just a few example methods that are used for measuring shear wave velocity, which is a key parameter for site response analysis. However, only the small-strain and linear behavior of soil is captured by these in situ tests [1].

The process of estimating site properties from recorded ground motions, which is called seismic inversion or site identification, has been an important topic of research (e.g., [11,12]). Seismic events can be regarded as in situ dynamic tests carried out by nature. The data recorded during these events offer opportunities for studying in situ soil behavior. However, because of the lack of nonlinear identification methods, so far most of the site identification studies have been limited to linear or equivalent linear properties of soil layers [13,14]. There are a few studies in which nonlinear soil behavior has been identified, but their applicability is limited because of the material model simplicity (e.g., [15,16]). Moreover, in many such studies, the in-depth (within) motions are used as input excitations with a conventional outcrop approach (absorbing boundary condition), which can be inaccurate or limiting because of wave reflection effects [17].

In this study, we propose a Bayesian model updating approach for nonlinear finite element (FE) model updating and site identification using seismic downhole array data. By considering one-dimensional (1D) site response and having general information such as soil layering and types, we develop, test, and validate two identification methods using geotechnical downhole array data: (1) an input-output (IO) identification method to estimate only the nonlinear soil properties using the measured incident (or within) motions and site responses and (2) an output-only (OO) identification method to jointly estimate the nonlinear soil properties and the incident motions by using the measured site responses. While the core algorithm used in this study is not new and Bayesian methods have been used for the estimation of soil parameters [18,19,20], the proposed solutions for estimating nonlinear soil properties, as well as incident motions from geotechnical data under various site conditions and instrumentation layouts, are novel. Although a specific nonlinear constitutive model is used in this study, the proposed approaches can be readily extended to consider the plausibility of other constitutive models.

## 2. Proposed Framework

### 2.1. Problem Definition

**Figure 1:**
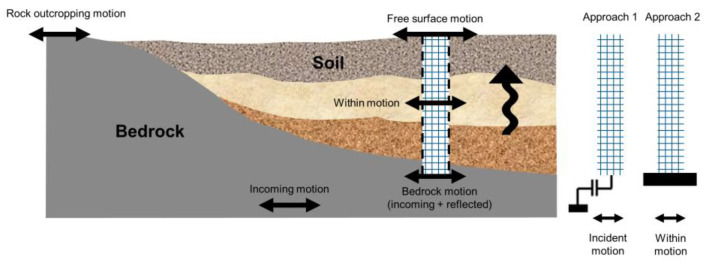
One-dimensional site analysis approaches.

Two approaches exist to carry out one-dimensional (1D) dynamic site response analyses, as shown in Figure 1. In the traditional approach (Approach 1), the site is modeled down to the bedrock depth (half space), where an absorbing boundary condition is modeled to prevent wave reflections. In this approach, the incident motion must be used as the input base excitation, which can be theoretically obtained from nearby rock outcrop motion. The accuracy of this approach depends on two key assumptions: (1) there is no impedance contrast below the boundary level, and (2) there is a nearby recorded rock outcrop motion. In the second approach (Approach 2), the domain can be cut at any depth as long as the so-called within motion (i.e., the measured in-depth motion) is used as the input excitation with a fixed boundary condition.

**Figure 2:**
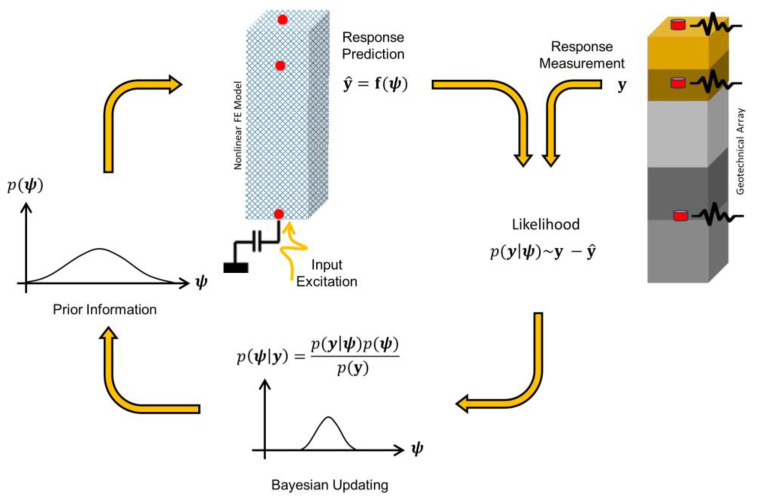
Sequential Bayesian estimation method for joint model and input identification (ψ) using finite element prediction ($\hat{y}$) and measured responses (y).

We propose to solve the inverse problem using an output-only sequential Bayesian estimation approach for nonlinear model updating [21,22,23], as shown in Figure 2. In this approach, the 1D FE model of the site is modeled down to the bedrock depth with an absorbing boundary condition if the bedrock is not rigid. A soil constitutive model is chosen, and its parameters are identified, along with the unknown incident motions through the Bayesian model updating approach, the details of which will be presented in the next section. In this study, the effects of modeling errors are neglected, so the presence of any modeling error, e.g., 2D effects, spatial variability, different number of layers, etc., could affect the identified nonlinear soil properties.

**Figure 3:**
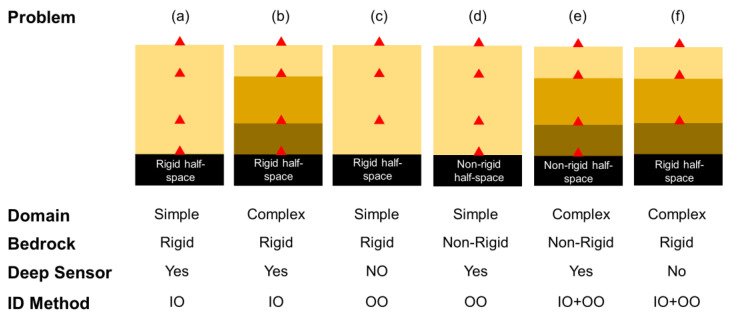
Various possible problem cases and proposed solution approaches. IO and OO represent the input-output and output-only identification methods, respectively.

The abovementioned joint input-parameter estimation solution is prone to identifiability issues and estimation uncertainties, especially if there are several unknown parameters, such as a layered site with unknown incident motion or limited in-depth recorded data. In addition, the computational cost significantly increases when the number of parameters increases. To resolve this issue, we classify five problem cases, as schematically shown in Figure 3, according to the bedrock condition, the domain complexity, and the sensor locations. The simplest case is the one with a few soil layers (simple domain), multiple in-depth measurements, a rigid bedrock, and a sensor at the bedrock (Figure 3a). In this case, we use the input-output (IO) version of the Bayesian model updating approach to estimate only the soil material parameters. If the domain has several soil layers (complex domain), but there is still a sensor at the bedrock, and the bedrock is rigid (Figure 3b), then the IO identification is still the best solution strategy. If the domain is simple, but there is no sensor at the bedrock (Figure 3c), then the output-only (OO) version of the Bayesian model updating approach can be used. The same strategy can be used for a simple domain with a nonrigid half space if there is a sensor at the nonrigid bedrock (Figure 3d). For more-complex cases (Figure 3e,f), we propose a two-step identification method. In the two-step method, the domain is assumed fixed at the deepest instrumented point, and the IO identification method is used to estimate the material parameters. Then, in the second step, the OO identification method is employed to estimate the remaining parameters (e.g., layers between half space and the deepest instrumented level) and the incident motions at the bedrock.

### 2.2. Bayesian Model Updating

In this section, the Bayesian finite element model updating formulation is presented for the general case of joint parameter-input estimation (OO identification method), while it can be easily reduced to the parameter-only estimation (IO identification method).

**Figure 4:**
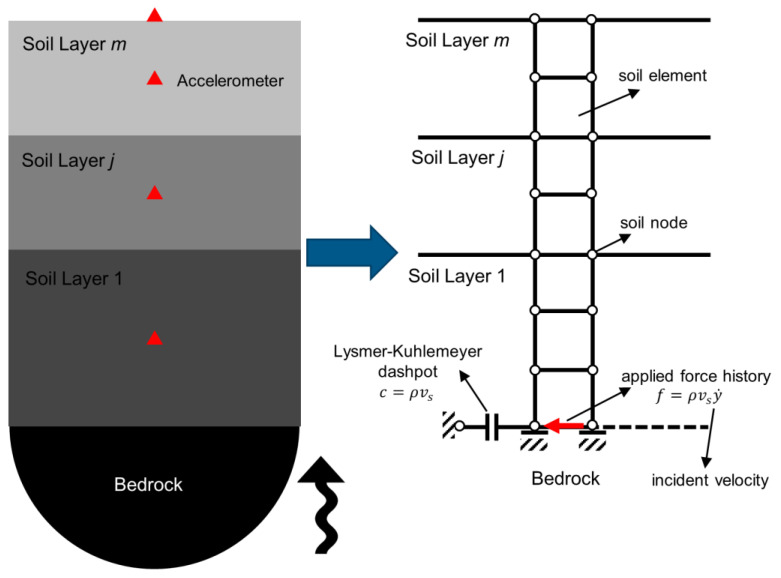
The generic site (**left**) and its finite element model (**right**).

Figure 4 (left) shows a generic site with m soil layers on top of an elastic (nonrigid) half space (bedrock). The seismic excitation is vertically propagating upward, and the absolute acceleration response of the site is measured at multiple points, which are not necessarily at the layer boundaries. The finite element model of this site is shown in Figure 4 (right), in which **the** Lysmer–Kuhlemeyer [24] dashpot is used to represent radiation damping provided by a semi-infinite nonrigid half space, and the seismic excitation is applied using a horizontal force time history calculated as the multiplication of the incident velocity motion and dashpot’s coefficient [25].

The nonlinear response of the FE model at each time step, i and ${\hat{y}}_{i}$, can be obtained as

(1) $${\hat{y}}_{i}=h_{i}\left(\theta ,f_{1:i}\right)$$

where $\theta =\left[\theta_{1},\ldots ,\theta_{n_{\theta }}\right]$ is the model parameter vector containing $n_{\theta }$ parameters (e.g., parameters of the material constitutive models) characterizing the FE model, $f_{1:i}$ is the time history of the applied force, and $h_{i}\left(.\right)$ is the response function encapsulating the dynamic of the model from the beginning to time step i. The difference between this predicted response, ${\hat{y}}_{i}$, and the measured response, $y_{i}$, can be represented by a noise model as

(2) $$v_{i}\left(\theta ,f_{1:i}\right)=y_{i}-{\hat{y}}_{i}\left(\theta ,f_{1:i}\right)$$

where $v_{i}\in R^{n_{y}\times 1}$ or the simulation error contains modeling, parameter, and measurement uncertainties. In this study, we neglect the effects of the modeling uncertainty and try to find the estimates of the unknown parameter vector, i.e., $\psi_{i}={\left[\theta^{T},f_{1:i}{}^{T}\right]}^{T}$, to minimize this simulation error. To do so, the simulation error is ideally modeled as an independent and stationary zero-mean Gaussian white noise process. Herein, we employ a sequential estimation approach for model updating. In this approach, the time domain is divided into successive overlapping windows, which will be called the estimation windows hereinafter, and the model updating problem is iteratively solved at each window to estimate the unknown parameter vector, and then it moves to the next window.

Assume that the m-th estimation window with length $t_{l}=t_{2}^{m}-t_{1}^{m}$ spans from $t_{1}^{m}$ to $t_{2}^{m}$. At this estimation window, the unknown parameter vector is defined as a $n_{\psi }\times 1$ vector $\psi_{m}={\left[\theta^{T},f_{t_{1}^{m}:t_{2}^{m}}^{m}{}^{T}\right]}^{T}$, where $\psi_{m}\in R^{\left(n_{\theta }+t_{l}\right)\times 1}$ and can be estimated by using a parameter-only Kalman filtering method (e.g., [26]). In this approach the evolution of the parameter vector is characterized through a random walk [27], i.e.,

(3) $$\psi_{m,k+1}=\psi_{m,k}+\gamma_{m,k}$$

Corresponding to the state equation above; a measurement equation can be defined as

(4) $$y_{t_{1}^{m}:t_{2}^{m}}={\hat{y}}_{t_{1}^{m}:t_{2}^{m},k+1}\left(\psi_{m,k+1}\right)+v_{t_{1}^{m}:t_{2}^{m},k+1}$$

where $\gamma_{m,k}\sim N\left(0,Q\right)$, $v_{t_{1}^{m}:t_{2}^{m},k+1}\sim N\left(0,\tilde{R}\right)$, and $\tilde{R}\in R^{\left(t_{l}\times n_{y}\right)\times \left(t_{l}\times n_{y}\right)}$ constitute a block diagonal matrix, whose block diagonals are the simulation error covariance matrix R. Here, Q, which is referred to as the process noise covariance matrix in Kalman filtering literature [27], is a diagonal matrix with small positive diagonal entries relative to the elements of matrix $P_{\psi ,m,k}^{+}$. At each estimation window, the estimation process is iteratively repeated, as depicted by the iteration number k, and the mean and covariance of the parameter vector are updated on the basis of the misfit between the measured and estimated time history responses.

In this study, an unscented Kalman filtering (UKF) [28] method is used to update the unknown parameter vector at each iteration. While other methods, such as sampling techniques (e.g., particle filters, Markov chain Monte Carlo) or ensemble-based Kalman filters, can be used for uncertainty propagation and posterior PDF estimation (see, e.g., [29,30,31,32,33]), the UKF is employed here to reduce the computational costs, which could be cumbersome for large-scale problems. To do so, the nonlinear FE model is evaluated in parallel at a set of $2n_{\psi }+1$ deterministically selected realizations of the unknown parameter vector around the prior mean estimate ${\hat{\psi }}^{-}$, which are called sigma points (SPs) and are denoted by $\vartheta^{j}$. Using these FE evaluations, the mean vector, $\bar{y}$, and covariance matrix, ${\hat{P}}_{yy}$, of the predicted responses, and the cross-covariance matrix of ψ and y, ${\hat{P}}_{\psi y}$, are computed using a weighted sampling method:

(5) $$\bar{y}=\sum_{j=1}^{2n_{\psi }+1}W_{m}^{j}{\hat{y}}_{i}\left(\vartheta^{j}\right)$$

(6) $${\hat{P}}_{yy}=\overset{2n_{\psi }+1}{\underset{j=1}{\sum }}W_{e}^{j}\left[{\hat{y}}_{i}\left(\vartheta^{j}\right)-\bar{y}\right]{\left[{\hat{y}}_{i}\left(\vartheta^{j}\right)-\bar{y}\right]}^{T}+R$$

(7) $${\hat{P}}_{\psi y}=\overset{2n_{\psi }+1}{\underset{j=1}{\sum }}W_{e}^{j}\left[\vartheta^{j}-{\hat{\psi }}^{-}\right]{\left[{\hat{y}}_{i}\left(\vartheta^{j}\right)-\bar{y}\right]}^{T}$$

where $W_{m}^{j}$ and $W_{e}^{j}$ denote weighting coefficients [28]. The UKF prediction-correction procedure is employed as follows to estimate the posterior parameter mean vector, ${\hat{\psi }}^{+}{}_{m,k+1}$, and the covariance matrix, ${\hat{P}}_{\psi ,m,k+1}^{+}$, at each iteration as

(8) $${\hat{\psi }}^{+}{}_{m,k+1}={\hat{\psi }}^{-}{}_{m,k+1}+K\;\left(y_{t_{1}^{m}:t_{2}^{m}}-\bar{y}\right)$$

(9) $$P_{\psi ,m,k+1}^{+}=P_{\psi ,m,k+1}^{-}-K\left({\hat{P}}_{yy}+\tilde{R}\right)K^{T}$$

where ${\hat{\psi }}^{-}{}_{m,k+1}={\hat{\psi }}^{+}{}_{m,k}$ and $P_{\psi ,m,k+1}^{-}=P_{\psi ,m,k}^{+}+Q$, and the Kalman gain matrix is calculated as

(10) $$K={\hat{P}}_{\psi y}{\left({\hat{P}}_{yy}\right)}^{-1}$$

This process continues at each iteration until a convergence, e.g., $\left|{\hat{\psi }}^{+}{}_{m,k+1}-{\hat{\psi }}^{+}{}_{m,k}\right|<0.02\times {\hat{\psi }}^{+}{}_{m,k-1}$, is achieved and the estimation process moves to the next window. While the size of the successive overlapping windows is not critical, it should not be too long or too short. A long time window contains a significant amount of information, which may result in divergence if the initial variance of parameters is large. On the other hand, a short window may not include significant information about the dynamic of the system. More information about the window-based estimation and the impacts of window length can be found in [34]. As a general guideline, a time window with an approximate length of 10 cycles of the first mode of the system is a good choice.

### 2.3. Material Model

Over the past few decades, various nonlinear soil constitutive models have been devised [35,36,37,38,39]. For example, on the basis of the multisurface concept soil plasticity, Elgamal and colleagues [39] developed a nonlinear soil model with a nonassociative flow rule to reproduce the well-known dilatancy effect. This model, in which the yield surface is defined on the basis of the Drucker–Prager [40], is frequently used in the direct soil-structure interaction (SSI) simulation problems and is available in OpenSees [41]. This model approximates the soil behavior within a broad range of strain regimes thanks to its multiple hierarchical yield surfaces, but a large number of requisite model parameters renders its calibration process formidable. In addition, the model may exhibit spurious sensitivities to these parameters. These two major drawbacks can be problematic in solving inverse problems using real-life data.

Borja and Amies [36] have proposed a model (called BA model hereinafter) with a simpler structure. This model has only a bounding surface and a vanishing elastic region, which can be defined by a few parameters and is favorable in calibration/inverse problems. This model has been successfully employed to reproduce the downhole array motions recorded at the Lotung site in Taiwan through one-dimensional nonlinear site response analysis [42]. A brief description of this model and the parameters that need to be adjusted are presented in the following, and further details can be found in the original reference [36] and its later extension [43].

The BA model admits an additive decomposition of the stress into inviscid (frictional), $\sigma^{inv}$, and viscous, $\sigma^{vis}$, parts as

(11) $$\sigma =\sigma^{inv}+\sigma^{vis}$$

where

(12) $$\sigma^{inv}=C^{e}:\left(\epsilon -\epsilon^{p}\right)$$

(13) $$\sigma^{vis}=D:\dot{\epsilon }$$

in which $C^{e}$ and D represent elastic stiffness and viscous-damping tensors, respectively; ϵ represents the total strain tensor; $\epsilon^{p}$ represents the plastic strain tensor; and $\dot{\epsilon }$ represents the total strain rate. In Equation (13), “:” denotes the double dot operator. Given this decomposition, the model can incorporate material-level strain-rate-dependent damping, which enables a modeler to match complex field-identified damping behavior [44,45].

Borja et al. [46] derived the following consistent tangent moduli to achieve an optimal rate of numerical convergence:

(14) $$C_{ep}^{inv}=\frac{d\sigma_{n+1}^{inv}}{d\epsilon_{n+1}}=K1⊗1+\gamma I_{dev}+\frac{\partial \gamma }{\partial \epsilon_{n+1}}⊗\Delta \epsilon^{\prime }$$

where the fourth-order deviatoric identity tensor is defined as $I_{dev}=I-\frac{1}{3}1⊗1$, I and 1 being the fourth- and second-order identify tensors, respectively, and ⊗ is the tensor product. In Equation (14), K is the bulk modulus and parameter γ is defined as $\Delta \sigma^{\prime }=\gamma \Delta \epsilon^{\prime }$, where $\Delta \sigma^{\prime }$ and $\Delta \epsilon^{\prime }$ are the deviatoric stress and strain increments, respectively. In this study, we use element-level stiffness-proportional damping to have more control over damping in the modeling, which is equivalent to having a viscous-damping tensor:

(15) $$D=a_{1}\;C^{e}$$

where $a_{1}$ is the stiffness-proportional damping coefficient. Therefore, using Equations (11) and (12) stress increment from viscous-damping contribution can be calculated as

(16) $$\sigma_{n+1}^{vis}=\frac{a_{1}}{dt}\;C^{e}:d\epsilon_{n+1}$$

in which dt is the time interval. Consequently, the total consistent tangent stiffness moduli is

(17) $$C_{ep}^{}=K1⊗1+\gamma I_{dev}+\frac{a_{1}}{dt}\;C^{e}$$

in which the effects of the unsymmetric part of the inviscid stiffness ($\frac{\partial \gamma }{\partial \epsilon_{n+1}}⊗\Delta \epsilon^{\prime }$) is excluded for the sake of computational efficiency.

The advantages of the BA model are clear: (1) it is a thermodynamically consistent model based on the bounding surface plasticity framework with well-defined parameters; (2) if calibrated correctly, the model can accurately predict soil behavior under multiaxial stress states; and (3) the number of parameters required to be set in the BA model is limited. These characteristics make the BA model an appealing choice for model-calibration applications. This study will not go into details here, for the sake of brevity (details can be found in [36]), but the relationship between the common $G/G_{max}$ (strain-dependent shear modulus to maximum/elastic shear modulus) curve and the parameters of the model is as follows:

(18) $$\frac{G}{G_{max}}=1-\frac{3}{2\tau_{0}}\int_{0}^{2\tau_{0}}{\left[h{\left(\frac{S_{u}+\tau_{0}-\tau }{\tau }\right)}^{m}+H_{0}\right]}^{-1}d\tau$$

where $G=\tau_{0}/\gamma_{0}$ represents the secant shear stiffness ($\tau_{0}$ is the stress at the maximum strain $\gamma_{0}$), $S_{u}$ represents the simple shear test soil strength, τ represents shear stress, and parameters h, m, and $H_{0}$ control the hardening behavior. As seen, there are only six parameters to completely define the soil model: $G_{max}$, $S_{u}$, h, m, $H_{0}$, and $a_{1}$. The contribution of $H_{0}$ is usually insignificant for typical soils, and in this study, it is assumed to be zero [43]. Therefore, in this paper, we consider only five parameters in the Bayesian model updating studies. Note that in some cases, a few of these parameters cannot be identifiable, because of the less sensitivity of the site response to those parameters. Although such parameters can be predetected for IO cases through sensitivity/identifiability analyses, we keep them in the list of updating parameters in all synthetic and real-life examples. The BA model has been successfully implemented in OpenSees and extensively verified and validated [47].

## 3. Verification Studies

**Figure 5:**
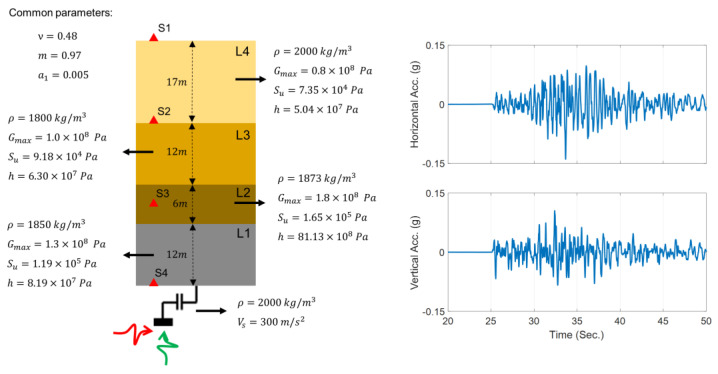
Soil profile used for the verification case studies (**left**) and horizontal and vertical incident motions (**right**).

Four problems are devised to verify the proposed methods, which cover most of the cases presented in Figure 3. Figure 5 (left) shows the profile of the studied site, which consists of four layers numbered from bottom to top (L1 to L4). This 47 m site is on top of an elastic half space. A 1D plane-strain FE model of the site is prepared in OpenSees using quad element types. The model mesh size is 1 m in the vertical direction, which can resolve frequencies of up to 40 Hz. In all case studies of this paper, the width of the elements is set to be the minimum vertical element size in the soil column. The half space at the bottom of the model is replaced by horizontal and vertical dashpots with coefficients $c_{h}=\rho V_{s}$ and $c_{v}=\rho V_{p}$, respectively, in which ρ is the mass density of the half space and $V_{s}$ and $V_{p}$ are, respectively, the shear and compressional wave velocity of the half space. Moreover, the parameters defining the nonlinear BA model of all four layers are shown in this figure. The nonlinear model is solved using a Newton method and integrated in time using the Hilber–Hughes–Taylor (HHT) [48] time integration method to find the site response at instrumented locations shown as S1 to S4. The effect of pore water pressure is neglected, and all analyses are carried out in the total stress state.

It is assumed that the measurements are collected using four accelerometers, as shown by red triangles in Figure 5 (left), which record the response of the site under two vertically propagating shear and compressional excitations shown in red and green, respectively. The vertical and north–south velocity motions recorded during the 2014 South Napa earthquake at 35 m depth at the CSMIP (California Strong Motion Instrumentation Program) [49] station #68323 are used as incident excitations. Both components are magnified by a factor of 10 to ensure that the site behaves nonlinearly, which results in an input excitation with a horizontal peak acceleration of 0.14 g. Figure 5 (right) shows the scaled vertical and horizontal accelerations of these two input excitations.

The verification case studies consist of simulation and estimation stages. In the simulation stage, the seismic responses of the nonlinear site are simulated at the instrumentation locations using the model parameters set at their baseline (or true) values indicated in Figure 5 (left). Random noises with 5% RMS (root mean squares) noise-to-signal ratio are added to the simulated responses to mimic measurement data. In the estimation stage, the measurement data are used for site identification or joint site and incident motion identification, as explained for each case below.

### 3.1. Case 1: Unknown Complex Domain with Known Incident Motion

In the first case study, it is assumed that the incident motion is available, such as through outcrop measurements. So the IO identification method is employed to estimate five unknown parameters ($G_{0},\;S_{u},\;h,\;m,\;a_{1}$) for each of the four layers (i.e., a total of 5 × 4 = 20 unknown parameters to be estimated). For this purpose, all the measurements of S1 to S4 are used as measured responses. Note that only horizontal measurements are used in this study, and although the vertical incident motion was used to simulate the site response, the vertical measurements are not used for the estimation. This case represents problems (a) and (b) of Figure 3. A 30% initial error in the model parameters is assumed with respect to their corresponding baseline (true) parameter values. The initial coefficient of variation (COV) for each parameter is selected as 10%. The filter parameters are set as $R=1\times 10^{-5}\;m^{2}/s^{4}\;I_{n_{y}\times n_{y}}$, $Q=1\times 10^{-14}\;I_{n_{\theta }\times n_{\theta }}$, and the estimation window length ($t_{l}$) is 1 s, which includes 100 time steps, and a 30% overlap is considered between successive estimation windows.

**Figure 6:**
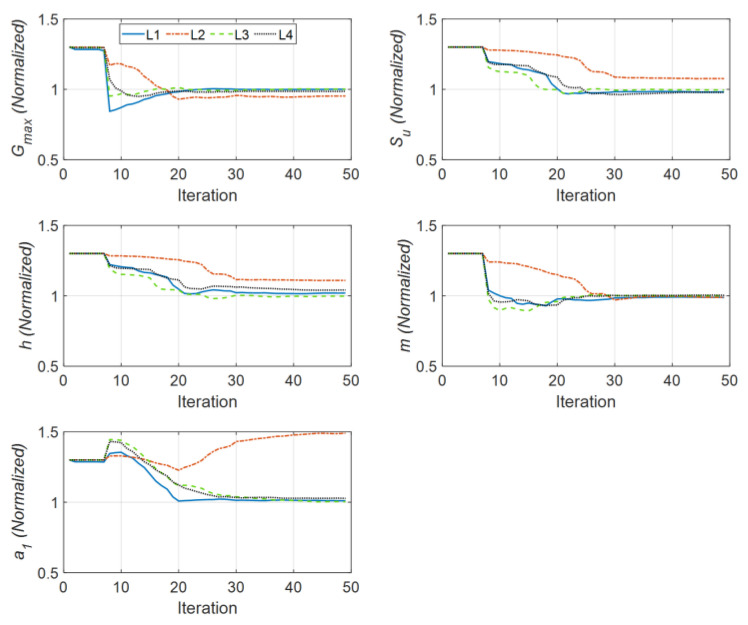
Identified parameters in Case Study 1 (normalized by the corresponding true values).

**Figure 7:**
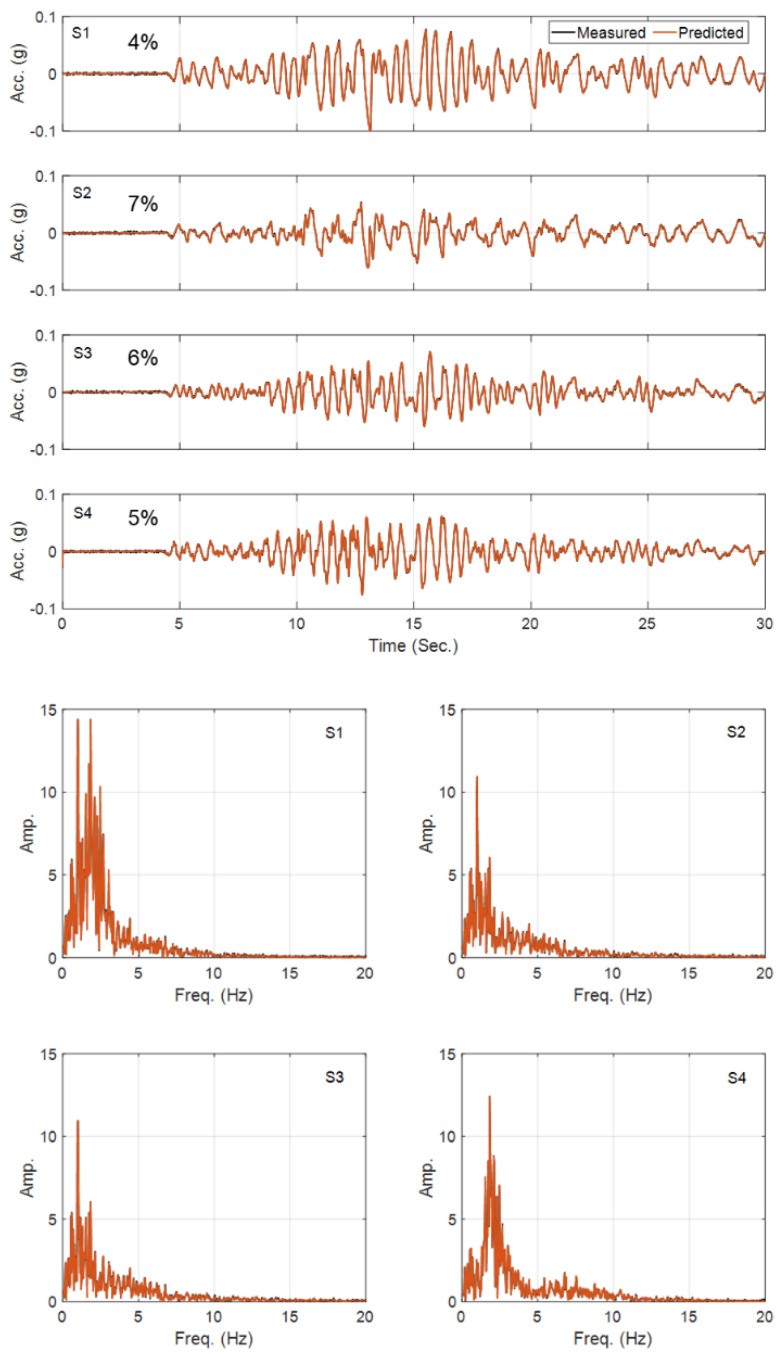
Comparison between the measured and predicted acceleration responses in Case Study 1 (top—time domain; bottom—frequency domain).

Figure 6 shows the history of the estimated parameters (normalized by their corresponding true values) at every estimation iteration. As seen in this figure, most of the parameters quickly converge to the true values. There are some errors in the identified parameters of the second layer, which is expected because its contribution to the response of the site is small because of its limited thickness compared with the other layers. Specifically, the Rayleigh damping parameter ($a_{1}$) of this layer has little effect on the measured responses. In support of this statement, see Figure 7, which shows a comparison between measured (simulated) acceleration responses at four instrumented levels with the corresponding predictions obtained using the final estimated parameters. As seen, the predicted responses are identical to the measured (simulated) responses in both time and frequency domains. As a quantitative measure, the relative root mean square errors (RRMSEs) of the predicted signals are also calculated, in Equation (19), and shown on each plot.

(19) $$RRMSE\left(\%\right)=\frac{\sqrt{\sum_{i=1}^{N}{\left({\hat{y}}_{i}-y_{i}\right)}^{2}}}{\sqrt{\sum_{i=1}^{N}y_{i}{}^{2}}}\times 100$$

In this equation, ${\hat{y}}_{i}$ and $y_{i}$ represent, respectively, predicted and measured (simulated) responses at time instant *i*, and N represents the total number of time samples. As seen, the RRMSE values are very small, showing the accuracy of the estimated model. Throughout this paper, all time-domain comparisons are supplied with the RRMSE values. For synthetic examples, an RRMSE value greater than 10% is considered large [21], while in real-life cases, RRMSE values of up to 50% can still be considered small [50]. Table 1 presents the final estimation error for all 20 parameters. As already observed in Figure 6, this table shows that all parameters are identified with high accuracy, except those of the second layer, especially the Rayleigh damping parameter.

### 3.2. Case 2: Known Complex Domain with Unknown Incident Motion

**Figure 8:**
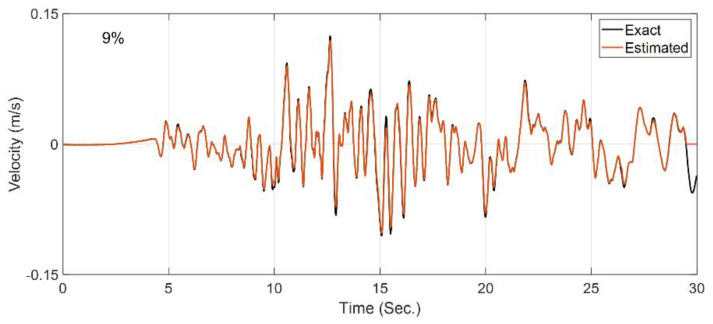
Comparison between the true incident motion and the estimated incident motion in Case Study 2.

In the second case study, it is assumed that the site is fully known, and the OO identification method is employed to back-calculate the horizontal incident motion by using the measurements at S1 to S4. This case is not directly representing the problems of Figure 3, but it shows the capability of the method for input estimation. Since the Bayesian model updating is prone to estimating spurious low-frequency input excitations [51], the velocity responses at the instrumented locations (S1 to S4) are also used, along with the acceleration responses, as measurements. A constant initial standard deviation of 0.1% m/s is assumed for all the unknown discrete values of the incident motion, which is used to construct the ${\hat{P}}_{f^{0}}$ matrix in Table 1. All the other filtering parameters are similar to the previous case study. Figure 8 shows the comparison between the exact (or baseline) incident motion and the estimated motion. As seen and quantitatively shown by the RRMSE value, the estimated motion is almost identical to the exact one, which verifies the OO identification method.

### 3.3. Case 3: Unknown Simple Domain with Unknown Incident Motion

**Figure 9:**
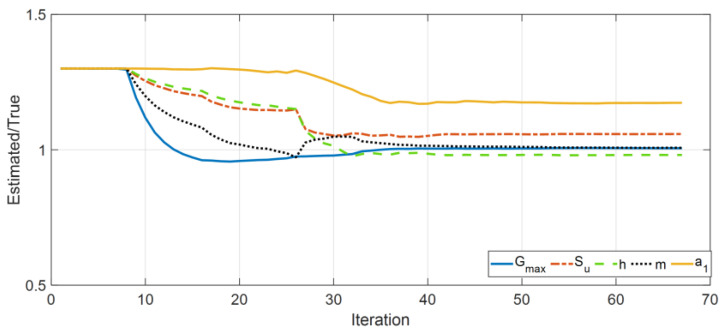
Identified parameters in Case Study 3 (normalized by the corresponding true values).

**Figure 10:**
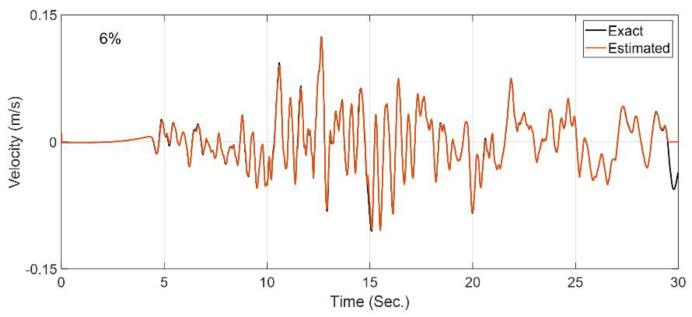
Comparison between the true incident motion and the estimated incident motion in Case Study 3.

**Figure 11:**
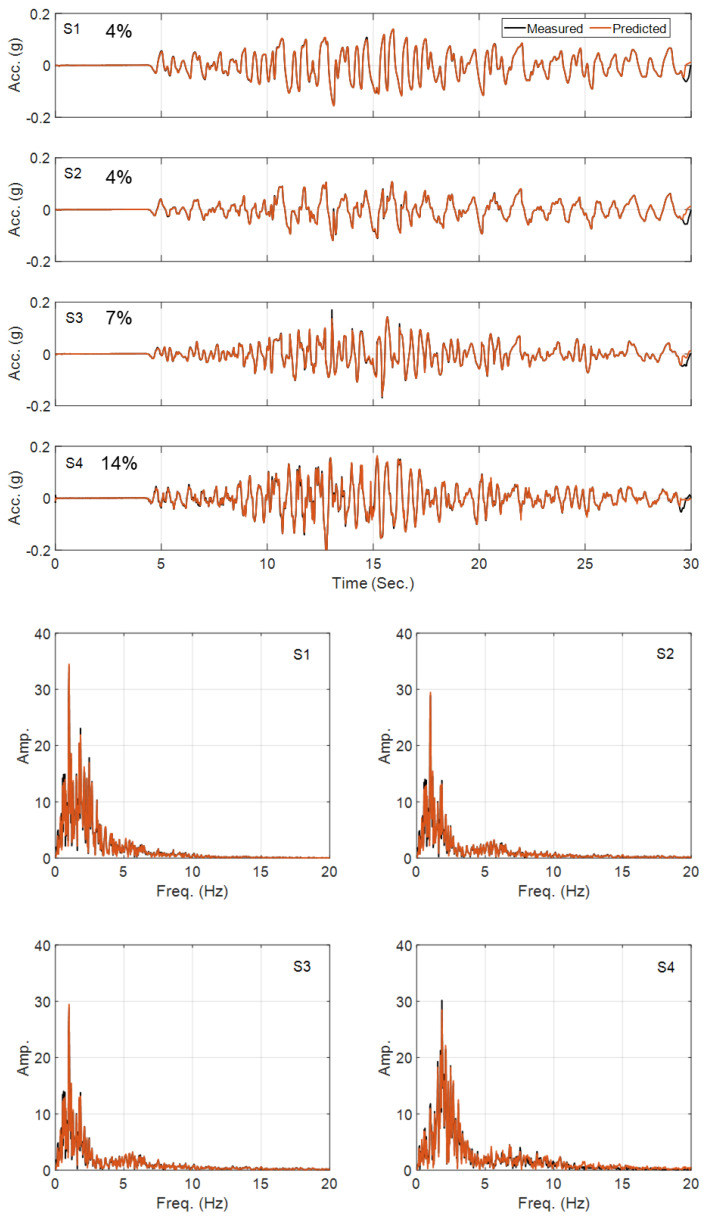
Time- and frequency-domain comparison between the measured and predicted acceleration responses in Case Study 3.

In the third case study, which represents problems (c) and (d) of Figure 3, the joint estimation of input and parameters is investigated. Here a joint input-parameter estimation (i.e., OO identification) is carried out for the simple domain. The domain is a uniform soil layer with the same thickness (47 m) as the total thickness of the complex domain with the baseline (true) values selected as $\rho =1850\frac{\mathrm{kg}}{m^{3}},\;G_{0}=1.3\times 10^{8}\;\mathrm{Pa},\;\nu =0.48,\;S_{u}=1.19\times 10^{5}\;\mathrm{Pa},\;h=8.19\times 10^{7}\;\mathrm{Pa},\;m=0.97,\;H_{0}=0,\;a_{0}=0,$ and $a_{1}=0.005$. The measurements at S1 to S4 are used for site identification, and the incident motion is unknown. Here the OO identification method is employed to estimate five model parameters ($G_{0}$, $S_{u}$, h, m, and $a_{1}$), where we assumed that the other parameters are fixed at their baseline values, and to estimate the horizontal incident motion by using measurements at S1 to S4. Other details are similar to the previous case studies. Figure 9 shows the history of parameter estimation (estimated parameters normalized by their corresponding true values) at every estimation iteration. As seen in this figure, $G_{0}$, h, and m converge to the true values, and there is a small error in $S_{u}$ and a large error in the estimation of $a_{1}$. The comparison between the estimated and exact (baseline) input excitation as well as measured and predicted acceleration responses shown respectively in Figure 10 and Figure 11 confirm that the response of the system is likely not sensitive to the incorrect estimation of $S_{u}$ and $a_{1}$. Final errors in the estimated parameters are reported in Table 2.

### 3.4. Case 4: Unknown Complex Domain with Unknown Incident Motion

The last case study is the most challenging one, in which the site is complex and contains many unknown parameters and an unknown incident motion, thus representing problems of Figure 3e,f. For this case, the identification method in its OO mode would not be useful. So a two-step identification method is used. In the first step, the domain is assumed to be fixed at the lowest instrumented level (S4) and the measured within motion (S4) is used as the input excitation to identify the 20 site parameters (four layers and five parameters per layer, as in Case Study 1). In the second step, the identified domain is used, and the horizontal component of the incident motion is estimated by setting the 20 site parameters at their estimated values. All the other details are similar to the previous case studies.

**Figure 12:**
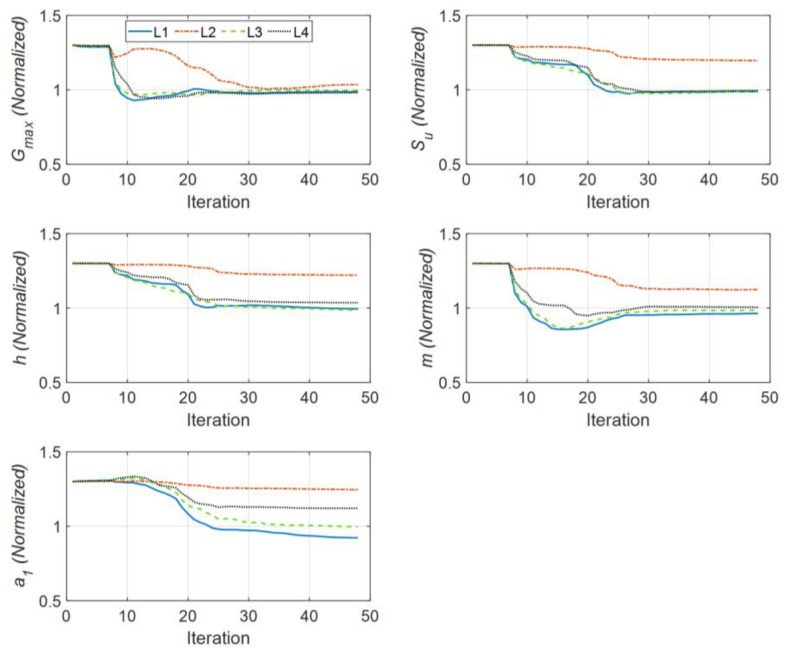
Identified parameters in Case Study 4 (normalized by the corresponding true values).

**Figure 13:**
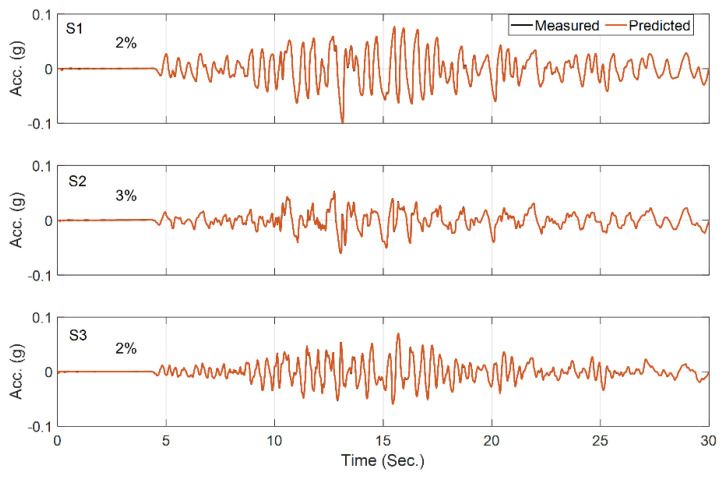
Comparison between the measured and predicted acceleration responses in Case Study 4.

Figure 12 shows the history of the estimated parameters (normalized by their corresponding true values) at every estimation iteration through the first step of the IO identification method. As seen, the results are quite similar to those obtained in Case Study 1. Figure 13 shows that the predicted responses at sensors S1 to S3 well match the measured responses (RRMSE values are very small). A comparison between the initial error and the final error of all 20 estimated parameters is shown in Table 3, which is quite similar to Table 1.

**Figure 14:**
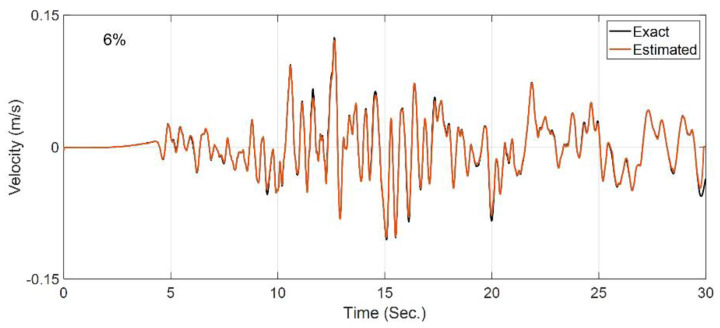
Comparison between the true incident motion and the estimated incident motion in Case Study 4.

Now, having identified the nonlinear parameters of all layers, the second step of the identification is carried out in the OO mode to estimate only the incident motion. In this step, the horizontal recording at S4 is included in the measurements. In addition, to reduce low-frequency error, both acceleration and velocity signals at the measurement locations are included in the measurements. Figure 14 shows the comparison between the exact (baseline) incident motion and the estimated incident motion. As seen, the estimated excitation is almost identical to the true incident velocity motion, with an RRMSE value of 6%. This case study verified the two-step identification approach.

## 4. Validation Studies

### 4.1. Centrifuge Test Data

**Figure 15:**
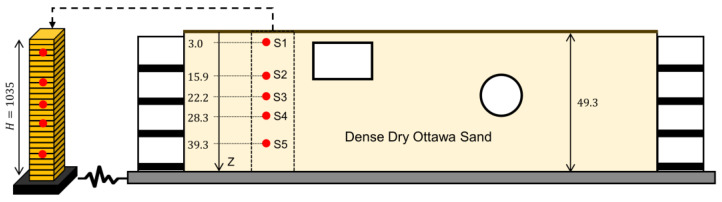
Validation case study using a centrifuge experiment: (**right**) soil domain and the instrumentation and (**left**) the FE model (all dimensions are in cm).

Centrifuge tests can provide precious data for validation studies because parameters of the domain and input excitations can be accurately measured. Here, we use data from a centrifuge test series on buried culvert structures. The test configurations are shown in Figure 15. The container is a flexible shear beam that is filled with dense dry Ottawa sand. Further details on these tests can be found in [52]. Several earthquake motions have been used to excite the base of the container under 21 g of centrifugal acceleration. Herein, the data from the free-field column (far from the structure) under the east–west component of the ground motion recorded in Santa Monica City Hall during the 1994 Northridge earthquake is used for a case study. The objective is to estimate the soil material parameters and the input excitation by using an OO model updating method for joint input-parameter estimation given that the domain is simple and has a limited number of unknown parameters.

A 1D FE model of the free-field column is developed in OpenSees using 0.4 m×0.4 m×0.4 m brick element, as shown in Figure 15 (left). The model consists of a H=10.35 m single layer with a BA material model with the following baseline parameters:

(20) $$\rho =1733\;\mathrm{kg}/m^{3}$$

(21) ν=0.3

(22) $$H_{0}=0$$

(23) $$a_{1}=P_{1}$$

(24) $$V_{s}=16.91+P_{3}{\left(\frac{z}{H}\right)}^{P_{2}}$$

(25) $$m=P_{4}$$

(26) $$h=\left[0.11+P_{5}{\left(\frac{z}{H}\right)}^{P_{6}}\right]G_{0}$$

(27) $$S_{u}=P_{7}G_{0}$$

where z represents the depth varying from zero at the surface to H at the base. Parameters $P_{1}$ to $P_{7}$ are considered as updating parameters with nominal values of 0.0032, 0.33, 193, 1.58, 0.47, 4.58, and 0.0015, respectively, which are very similar to the values reported in [53]. The model is fixed at the base, where a horizontal input excitation is applied. Other modeling details and assumptions are similar to those described for the verification case studies in Section 3.

**Figure 16:**
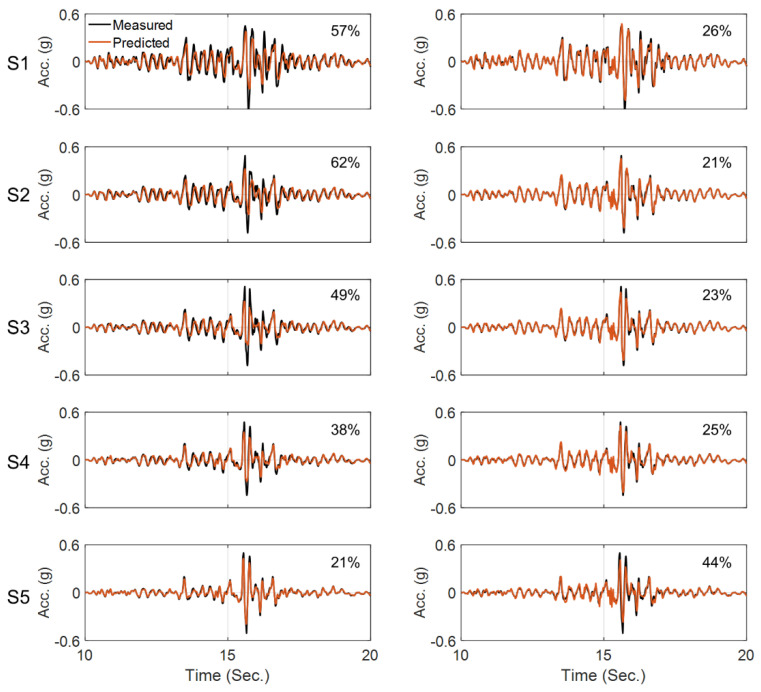
Comparison between recorded and predicted acceleration responses at five top sensors using baseline (**left**) and identified (**right**) model parameters.

**Figure 17:**
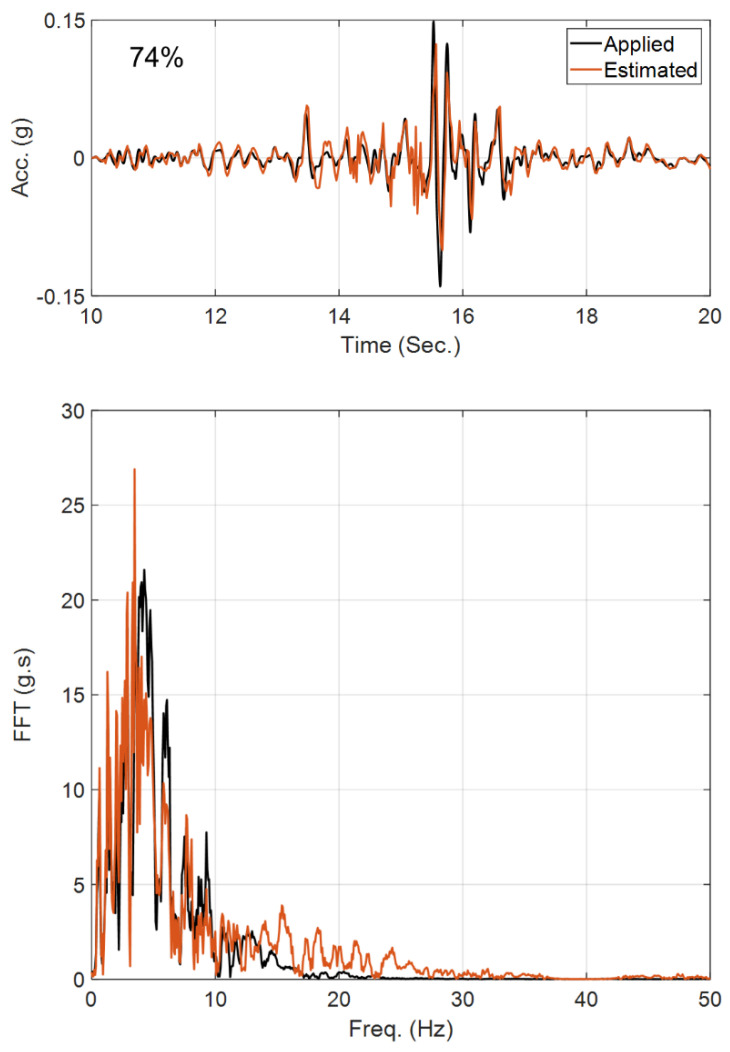
Comparison between the recorded and estimated input excitation in time (**top**) and frequency (**bottom**) domains.

We start the updating process assuming a 50% initial error in the parameter values with respect to their baseline values reported in [53]. The final errors of the estimated parameters with respect to their baseline parameter values are shown in Table 4. As seen, while there are some large differences between estimated values in this study and those reported in [53], the responses obtained from the updated model here better match the recorded responses (lower RRMSE values) than do those presented in [53], which are repeated in Figure 16. In this figure, acceleration responses recorded during the test are shown in black, and those predicted by the baseline [53] and updated parameters are shown in red in the left and right columns, respectively. Furthermore, the estimated base acceleration from the OO identification method is compared with the recorded motion in both time and frequency domains in Figure 17. The reasonable agreement between the measured and predicted responses in Figure 16 (right), along with the good agreement between the estimated base acceleration and the measured base acceleration, validates the accuracy of the OO identification method carried out here.

### 4.2. Seismic Data from Lotung Site

Located in the northeast of Taiwan, the Lotung large-scale seismic test (LSST) site was established in a seismically active region in 1985 to study seismic soil-structure-interaction (SSI) effects on nuclear power plants. In addition to the scaled structures constructed by the Electric Power Research Institute (EPRI) and the Taiwan Power Company [54], the ground was instrumented at the surface in several locations and at different depths, and the recorded data from this site have been the subject of many studies [42,55,56,57,58,59]. Among these studies, Zeghal and Elgamal [12,16] have extensively studied 18 earthquake datasets recorded between 1985 and 1986 (see Table 1 in [12,16]; data are publicly available at http://soilquake.net/Downholearray/Lotung/ (accessed on 8 December 2022)). Out of these 18 events, the respective amplitude resolutions of the signals in several events (Events 5, 6, 8, 9, 10, 15, 17, and 18) are not appropriate for identification studies. In addition, data on the first three events and Event 13 are not available. From the remaining sets, those with strong motion recordings are more suitable for the present study to observe soil nonlinearity. In addition, the source of the earthquake should be far from the site to make sure the site response is dominated by a 1D response. Given these criteria, Events 7 and 16, which have also been used by Borja et al. [42,43,59], are the best candidates. In this study, data from Event 7 are used because one of the sensors malfunctioned during Event 16.

**Figure 18:**
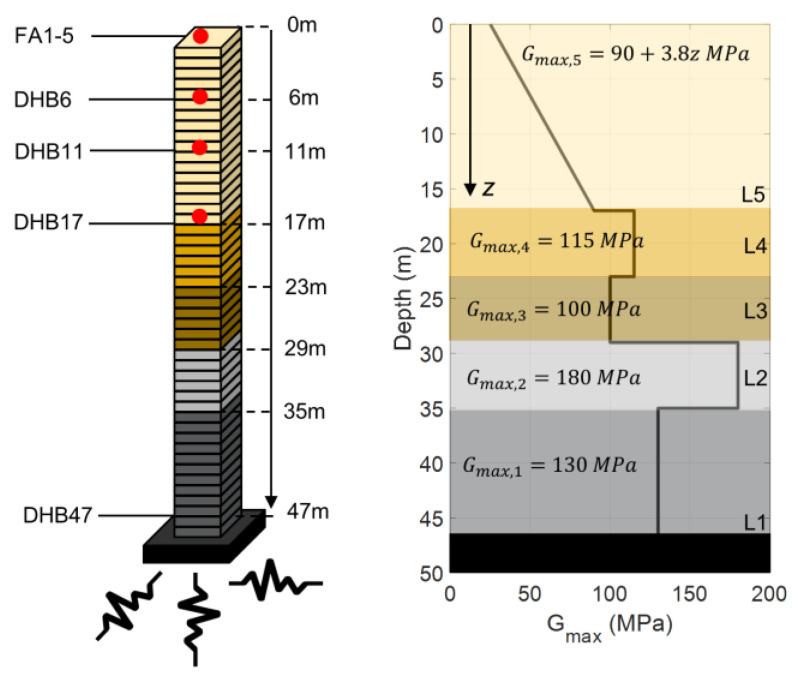
The FE model of the first 47 m of the Lotung site with a fixed bottom condition (**left**) and elastic shear modulus profile (**right**) (data to create the curve are from [43]).

The Lotung site is modeled using the information provided by Borja et al. [42,59] on the basis of the BA model. Figure 18 (left) shows the layered FE model of the first 47 m of the Lotung site, which includes instrumented depths at 0, 6, 11, 17, and 47 m (channels FA1-5, DHB6, DHB11, DHB17, and DHB47, respectively). The model is fixed at the lowest instrumented level (within boundary conditions) to be able to use measured data at DHB47 as input excitation for IO identification. The profile of the elastic shear modulus, $G_{max}$, of the site is shown in Figure 18 (right) [43]. The mass density is approximately $1800\;\mathrm{Kg}/m^{3}$ for the entire 47 m soil profile. According to this soil profile, the FE model includes five layers, in which the baseline parameters of the BA model for each layer are taken from [43] as $S_{u}=0.0011G_{max},\;H_{0}=0,\;h=0.63G_{max},\;m=0.97$, ν=0.48, and $a_{1}=0.005$, where $G_{max,i}$ = 130, 180, 100, and 115 for i=1 to 4, respectively, and $G_{max,5}=90+3.8z$, where z is the soil depth, as shown in Figure 18 (right). Note that because $S_{u}$ and h are functions of $G_{max}$, their values are different for each layer. The FE modeling details are similar to those used for the centrifuge case study in Section 4.1, but with the element size of 1 m, through which frequencies up to 25 Hz can be resolved.

For this case study, an IO identification method is used to estimate only the parameters of the soil layers, where we considered the measured excitation at the 47 m depth (sensor DHB47) as the incident motion. Furthermore, the measurement at 11 m depth (Sensor DHB11) is excluded from the measurement data used for IO identification and is used for the cross-validation of the updated model. In total, nine unknown parameters ($P_{1}$ to $P_{9}$) are selected in this study for model updating. Five parameters ($P_{1}$ to $P_{5}$) define the maximum shear modulus ($G_{max}$) of the five soil layers. Four remaining parameters ($P_{6}$ to $P_{9}$) define the other nonlinear properties of soil layers by using their corresponding $G_{max}$, as follows: $S_{u,i}=P_{6}G_{max,i}$, $h_{i}=P_{7}G_{max,i}$, $m_{i}=P_{8}$, and $a_{1,i}=P_{9}$. These parameters and their baseline values, recommended by Borja et al. [43], are shown in Table 5. It is assumed that ν=0.48 and $H_{0}=0$ for all layers.

**Figure 19:**
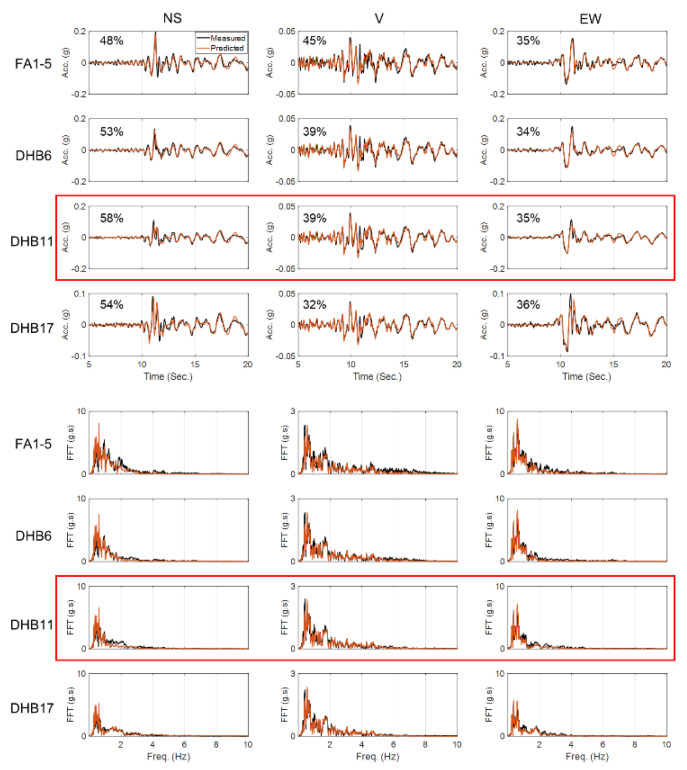
Time-domain (**top**) and frequency-domain (**bottom**) comparison between the recorded and predicted acceleration responses at four depths and in three directions. The recorded responses at depth 11 m (DHB11) are not used in the model updating and are here retained for cross-validation.

We started model updating with initial values 30% higher than the values recommended by Borja et al. [43]. The estimated final error in model parameter values relative to the baseline values are reported in Table 5. As this table shows, except for a few parameters, the results are not too far from the values recommended by Borja et al. [43]. Figure 19 shows a comparison between recorded acceleration responses at four depths and in three directions and those obtained using the updated model and identified parameters. Given that this is a real-life case study and there could be various sources of modeling errors, the level of agreement between the predicted and recorded responses in time and frequency domains is acceptable. In addition, the notable agreement between the recorded and predicted response at 11 m depth (Sensor DHB11) proves the reliability of the identification results.

## 5. Application to a Blind Site

**Figure 20:**
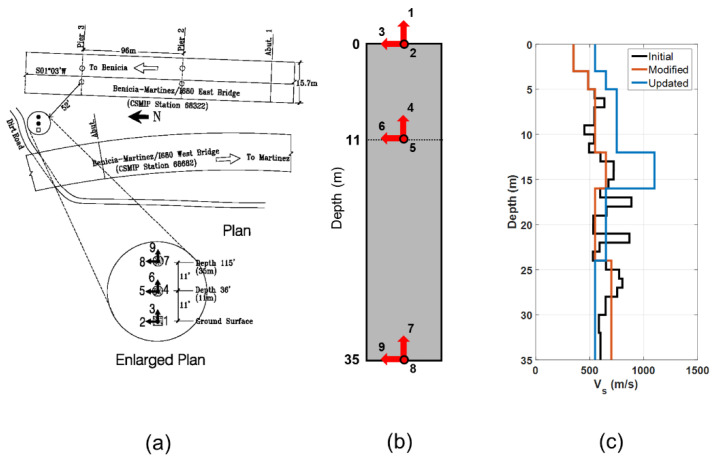
Benicia–Martinez geotechnical array. Instrumentation layout from CESMD (**a**), the location of sensors along the depth of the site (**b**), and shear wave velocity profile (**c**).

As a real-life application, the data obtained from CSMIP station #68323, located at the south end of the Benicia–Martinez bridge in California (latitude 38.0334 N, longitude 122.1170 W), are used. The site is composed of sediments underlain by slightly more-competent rock. Figure 20a shows the instrumentation layout. As shown in Figure 20b, the site is instrumented at the surface depth, −11 m, and at the −35 m depth, with triaxial accelerometers. The idealized version of the Vs profile was taken from [60] and is shown in Figure 20c, in black. This idealized profile was later refined (simplified) into six layers, as shown in Figure 20c, in blue. By the time of this study, eight earthquake events had been recorded at this station, which were all related to small events. The largest event was the 2014 South Napa event, which had a peak ground acceleration of about 0.03 g.

**Figure 21:**
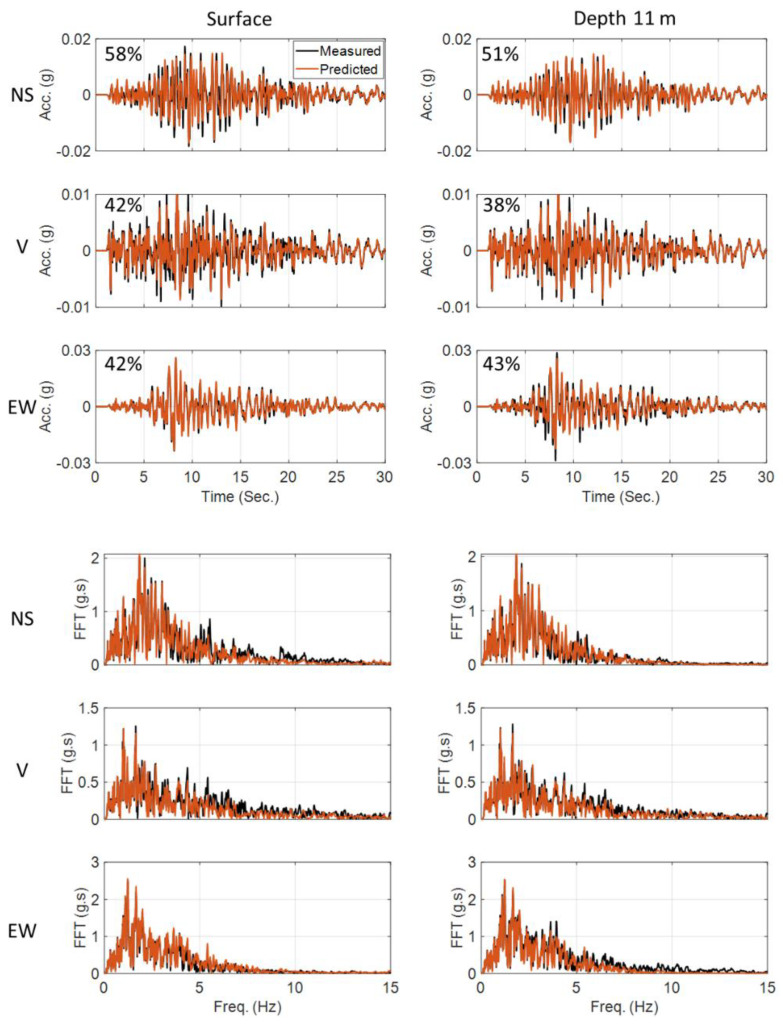
Time- and frequency-domain comparison between the recorded and estimated acceleration responses for the South Napa 2014 earthquake.

The Vs profile below 35 m is not known, so we carry out only the IO identification method, similar to what was conducted for the Lotung site in the previous section. The FE model includes the aforementioned six layers discretized with 1 m size elements, and it is fixed at the bottom. Five parameters at each layer ($G_{0},\;S_{u},\;h,\;m,\;a_{1}$) are considered as unknown model parameters, which result in 30 updating parameters in total. The initial values of these parameters are set using similar relationships as the Lotung site, i.e., $S_{u}=0.0011G_{0},\;h=0.63G_{0},\;m=0.97,\;\mathrm{and}\;a_{1}=0.0002$, where $G_{0}$ is calculated using the simplified $V_{s30}$ profile (Figure 20c) and mass density reported in [60]. Channels 7, 8, and 9 are used as input excitation, whereas Channels 2, 3, 5, and 6 are used as measured (output) data. Figure 21 shows the comparison between the predicted responses using the updated model and the recorded signals, which demonstrates the promising performance of the updated model in both time and frequency domains. The updated shear wave velocity profile is shown in Figure 20c, in red [60]. These results show that the measured Vs profile by [60] is relatively accurate, although it is smaller than what we obtained using earthquake data for the top four layers.

## 6. Discussion

The study presented the application of a Bayesian inference method based on unscented Kalman filters for nonlinear site identification by using recorded downhole array seismic data. In this process, the site of interest was modeled using a one-dimensional finite element model with nonlinear soil material constitutive models. Through the model updating process, the recorded downhole array data were utilized to estimate the parameters of the soil model as well as the input excitations (including incident, bedrock, and within motions). Four site identification problem setups, including simple and complex sites with various instrumentation scenarios, were considered and discussed in the paper. The site identification approach was first verified for these different setups using numerically simulated case studies. Then, two validation case studies were considered. In the first validation study, the free-field data from a centrifuge test on buried culvert structures were used in an output-only identification method to estimate jointly the model parameters and input excitation. The estimated input excitation was in close agreement with the measured input motion, which validated the proposed method. In the second validation study, real earthquake data recorded in the well-known Lotung site were used to estimate nonlinear soil properties in an input-output identification method, i.e., to estimate only the soil model parameters. The updated model was able to accurately predict recorded response motions at various sensor locations, including the one that was not used as a measurement in the model updating process. Finally, the site inversion approach was applied to the Benicia–Martinez site in California. Data recorded during the 2014 South Napa earthquake were used to identify the nonlinear properties of the site idealized in six layers using an input-output identification method. The results showed a promising application of the proposed site inversion approach to identify material parameters of the in situ soil by using recorded downhole array data.

## Figures and Tables

**Table 1:** The initial and final error of estimation parameters for Case Study 1.

Layer	Parameter	Initial Error (%)	Final Error (%)
1	$G_{0}$	+30	0.04
	$S_{u}$	+30	−1.86
	h	+30	1.97
	m	+30	−1.16
	$a_{1}$	+30	1.04
2	$G_{0}$	+30	−4.72
	$S_{u}$	+30	7.64
	h	+30	10.91
	m	+30	−1.09
	$a_{1}$	+30	49.02
3	$G_{0}$	+30	0.24
	$S_{u}$	+30	−0.42
	h	+30	−0.19
	m	+30	0.02
	$a_{1}$	+30	0.32
4	$G_{0}$	+30	−1.40
	$S_{u}$	+30	−2.15
	h	+30	4.08
	m	+30	0.35
	$a_{1}$	+30	2.72

**Table 2:** The initial and final error of estimation parameters for Case Study 3.

Layer	Parameter	Initial Error (%)	Final Error (%)
1	$G_{0}$	+30	0.56
	$S_{u}$	+30	5.80
	h	+30	−1.92
	m	+30	0.71
	$a_{1}$	+30	17.33

**Table 3:** The initial error and final error of estimation parameters for Case Study 4.

Layer	Parameter	Initial Error (%)	Final Error (%)
1	$G_{0}$	+30	−1.41
	$S_{u}$	+30	−1.17
	h	+30	−0.52
	m	+30	−3.63
	$a_{1}$	+30	−7.73
2	$G_{0}$	+30	3.48
	$S_{u}$	+30	19.69
	h	+30	22.09
	m	+30	12.4
	$a_{1}$	+30	24.59
3	$G_{0}$	+30	−0.21
	$S_{u}$	+30	−1.06
	h	+30	−1.32
	m	+30	−1.53
	$a_{1}$	+30	−0.35
4	$G_{0}$	+30	−1.86
	$S_{u}$	+30	−0.51
	h	+30	3.54
	m	+30	0.55
	$a_{1}$	+30	−12.09

**Table 4:** The baseline value and initial and final errors of estimation parameters for the centrifuge experiment case study.

Parameter	Baseline Values	Initial Error (%)	Final Error (%)
$P_{1}$	0.0032	+50	−1.4%
$P_{2}$	0.33	+50	+91%
$P_{3}$	193 (m/s)	+50	+50%
$P_{4}$	1.58	+50	+7%
$P_{5}$	0.47	+50	+800%
$P_{6}$	4.56	+50	−33%
$P_{7}$	0.0015	+50	−13%

**Table 5:** The baseline values and initial and final errors of the parameters for the Lotung case study.

Parameter	Baseline Value	Initial Error (%)	Final Error (%)
$G_{max,1}\;\left(\mathrm{MPa}\right)$	130 (MPa)	+30	−9
$G_{max,2}\;\left(\mathrm{MPa}\right)$	180 (MPa)	+30	111
$G_{max,3}\;\left(\mathrm{MPa}\right)$	100 (MPa)	+30	43
$G_{max,4}\;\left(\mathrm{MPa}\right)$	115 (MPa)	+30	−18
$G_{max,5}\;\left(\mathrm{MPa}\right)$ *	25 (MPa)	+30	−29
$S_{u,i}/G_{max,i}$	0.0011	+30	−36
$h_{i}/G_{max,i}$	0.63	+30	−39
$m_{i}$	0.97	+30	−41
$a_{1,i}$	0.005	+30	36

* Shear modulus of the fifth layer is assumed to linearly increase from this value with a slope of 3.8 GPa/m.

## Data Availability

All data and models presented in this manuscript are available from the corresponding author upon reasonable request.
